# Erratum to: *BMC Public Health*, Vol. 18

**DOI:** 10.1186/s12889-017-4709-6

**Published:** 2017-09-22

## Erratum

An error occurred during the publication of a number of articles in BMC Public Health. These articles were erroneously published in volume number 18, which is listed with a publication date of 2018. However, these articles were published in final form in the year 2017.

Provided below are the actual publication dates and the correct citation for each affected article. Please note that the citation refers to volume number 18 with the year as 2018.

Article: https://bmcpublichealth.biomedcentral.com/articles/10.1186/s12889-017-4535-x

Publication date: 10 July 2017.

Correct citation: Otu A, Ameh S, Osifo-Dawodu E, Alade E, Ekuri S, Idris J. An account of the Ebola virus disease outbreak in Nigeria: implications and lessons learnt. BMC Public Health. 2017;18:1. doi: 10.1186/s12889-017-4535-x

-

Article: https://bmcpublichealth.biomedcentral.com/articles/10.1186/s12889-017-4524-0

Publication date: 10 July 2017.

Correct citation: Tucktuck M, Ghandour R, Abu-Rmeileh N. Waterpipe and cigarette tobacco smoking among Palestinian university students: a cross-sectional study. BMC Public Health. 2017;18:1. doi: 10.1186/s12889-017-4524-0

-

Article: https://bmcpublichealth.biomedcentral.com/articles/10.1186/s12889-017-4536-9

Publication date: 10 July 2017.

Correct citation: Nahimana M, Nyandwi A, Muhimpundu M, Olu O, Condo J, Rusanganwa A et al. A population-based national estimate of the prevalence and risk factors associated with hypertension in Rwanda: implications for prevention and control. BMC Public Health. 2017;18:1. doi: 10.1186/s12889-017-4536-9

-

Article: https://bmcpublichealth.biomedcentral.com/articles/10.1186/s12889-017-4554-7

Publication date: 11 July 2017.

Correct citation: James M, Christian D, Scott S, Todd C, Stratton G, McCoubrey S et al. Active children through individual vouchers – evaluation (ACTIVE): protocol for a mixed method randomised control trial to increase physical activity levels in teenagers. BMC Public Health. 2017;18:1. doi: 10.1186/s12889-017-4554-7

-

Article: https://bmcpublichealth.biomedcentral.com/articles/10.1186/s12889-017-4565-4

Publication date: 11 July 2017.

Correct citation: Chourasia M, Raghavendra K, Bhatt R, Swain D, Dutta G, Kleinschmidt I. Involvement of Mitanins (female health volunteers) in active malaria surveillance, determinants and challenges in tribal populated malaria endemic villages of Chhattisgarh, India. BMC Public Health. 2017;18:1. doi: 10.1186/s12889-017-4565-4

-

Article: https://bmcpublichealth.biomedcentral.com/articles/10.1186/s12889-017-4553-8

Publication date: 11 July 2017.

Correct citation: Ha A, Lonsdale C, Lubans D, Ng J. Increasing students’ physical activity during school physical education: rationale and protocol for the SELF-FIT cluster randomized controlled trial. BMC Public Health. 2017;18:1. doi: 10.1186/s12889-017-4553-8

-

Article: https://bmcpublichealth.biomedcentral.com/articles/10.1186/s12889-017-4513-3

Publication date: 11 July 2017.

Correct citation: MacPhail C, Khoza N, Selin A, Julien A, Twine R, Wagner R et al. Cash transfers for HIV prevention: what do young women spend it on? Mixed methods findings from HPTN 068. BMC Public Health. 2017;18:1. doi: 10.1186/s12889-017-4513-3

-

Article: https://bmcpublichealth.biomedcentral.com/articles/10.1186/s12889-017-4561-8

Publication date: 11 July 2017.

Correct citation: Yeung J, Zhang Z, Kim T. Volunteering and health benefits in general adults: cumulative effects and forms. BMC Public Health. 2017;18:1. doi: 10.1186/s12889-017-4561-8

-

Article: https://bmcpublichealth.biomedcentral.com/articles/10.1186/s12889-017-4552-9

Publication date: 11 July 2017.

Correct citation: Kesten J, Bhattacharya A, Ashiru-Oredope D, Gobin M, Audrey S. The Antibiotic Guardian campaign: a qualitative evaluation of an online pledge-based system focused on making better use of antibiotics. BMC Public Health. 2017;18:1. doi: 10.1186/s12889-017-4552-9

-

Article: https://bmcpublichealth.biomedcentral.com/articles/10.1186/s12889-017-4548-5

Publication date: 11 July 2017.

Correct citation: Kushnir V, Sproule B, Cunningham J. Impact of large-scale distribution and subsequent use of free nicotine patches on primary care physician interaction. BMC Public Health. 2017;18:1. doi: 10.1186/s12889-017-4548-5

-

Article: https://bmcpublichealth.biomedcentral.com/articles/10.1186/s12889-017-4551-x

Publication date: 11 July 2017.

Correct citation: Steenbock B, Zeeb H, Rach S, Pohlabeln H, Pischke C. Design and methods for a cluster-controlled trial conducted at sixty-eight daycare facilities evaluating the impact of “JolinchenKids – Fit and Healthy in Daycare”, a program for health promotion in 3- to 6-year-old children. BMC Public Health. 2017;18:1. doi: 10.1186/s12889-017-4551-x

-

Article: https://bmcpublichealth.biomedcentral.com/articles/10.1186/s12889-017-4559-2

Publication date: 12 July 2017.

Correct citation: Tønnesen R, Hovind P, Jensen L, Schwarz *P. erratum* to: determinants of vitamin D status in young adults: influence of lifestyle, sociodemographic and anthropometric factors. BMC Public Health. 2017;18:1. doi: 10.1186/s12889-017-4559-2

-

Article: https://bmcpublichealth.biomedcentral.com/articles/10.1186/s12889-017-4571-6

Publication date: 12 July 2017.

Correct citation: Oshio T, Umeda *M. erratum* to: gender-specific linkages of parents’ childhood physical abuse and neglect with children’s problem behaviour: evidence from Japan. BMC Public Health. 2017;18:1. doi: 10.1186/s12889-017-4571-6

-

Article: https://bmcpublichealth.biomedcentral.com/articles/10.1186/s12889-017-4555-6

Publication date: 12 July 2017.

Correct citation: Den Broeder L, Uiters E, Hofland A, Wagemakers A, Schuit A. Local professionals’ perceptions of health assets in a low-SES Dutch neighbourhood: a qualitative study. BMC Public Health. 2017;18:1. doi: 10.1186/s12889-017-4555-6

-

Article: https://bmcpublichealth.biomedcentral.com/articles/10.1186/s12889-017-4564-5

Publication date: 13 July 2017.

Correct citation: Szostak-Węgierek D, Waśkiewicz A, Piotrowski W, Stepaniak U, Pająk A, Kwaśniewska M et al. Metabolic syndrome and its components in Polish women of childbearing age: a nationwide study. BMC Public Health. 2017;18:1. doi: 10.1186/s12889-017-4564-5

-

Article: https://bmcpublichealth.biomedcentral.com/articles/10.1186/s12889-017-4580-5

Publication date: 14 July 2017.

Correct citation: Qian L, Zhang F, Newman I, Shell D, Du W. Effects of selected socio-demographic characteristics on nutrition knowledge and eating behavior of elementary students in two provinces in China. BMC Public Health. 2017;18:1. doi: 10.1186/s12889-017-4580-5

-

Article: https://bmcpublichealth.biomedcentral.com/articles/10.1186/s12889-017-4575-2

Publication date: 14 July 2017.

Correct citation: Srivastava A, Singh D, Montagu D, Bhattacharyya S. Putting women at the center: a review of Indian policy to address person-centered care in maternal and newborn health, family planning and abortion. BMC Public Health. 2017;18:1. doi: 10.1186/s12889-017-4575-2

-

Article: https://bmcpublichealth.biomedcentral.com/articles/10.1186/s12889-017-4534-y

Publication date: 14 July 2017.

Correct citation: Fredriksson M, Eriksson M, Tritter J. Who wants to be involved in health care decisions? Comparing preferences for individual and collective involvement in England and Sweden. BMC Public Health. 2017;18:1. doi: 10.1186/s12889-017-4534-y

-

Article: https://bmcpublichealth.biomedcentral.com/articles/10.1186/s12889-017-4578-z

Publication date: 14 July 2017.

Correct citation: Verver S, Kapata N, Simpungwe M, Kaminsa S, Mwale M, Mukwangole C et al. Feasibility of district wide screening of health care workers for tuberculosis in Zambia. BMC Public Health. 2017;18:1. doi: 10.1186/s12889-017-4578-z

-

Article: https://bmcpublichealth.biomedcentral.com/articles/10.1186/s12889-017-4588-x

Publication date: 14 July 2017.

Correct citation: Bethge M, Mattukat K, Fauser D, Mau W. Rehabilitation access and effectiveness for persons with back pain: the protocol of a cohort study (REHAB-BP, DRKS00011554). BMC Public Health. 2017;18:1. doi: 10.1186/s12889-017-4588-x

-

Article: https://bmcpublichealth.biomedcentral.com/articles/10.1186/s12889-017-4567-2

Publication date: 14 July 2017.

Correct citation: Henry K, Swiecki-Sikora A, Stroup A, Warner E, Kepka D. Area-based socioeconomic factors and Human Papillomavirus (HPV) vaccination among teen boys in the United States. BMC Public Health. 2017;18:1. doi: 10.1186/s12889-017-4567-2

-

Article: https://bmcpublichealth.biomedcentral.com/articles/10.1186/s12889-017-4566-3

Publication date: 17 July 2017.

Correct citation: Fei F, Zhong J, Yu M, Gong W, Wang M, Pan J et al. Impact of injury-related mortality on life expectancy in Zhejiang, China based on death and population surveillance data. BMC Public Health. 2017;18:1. doi: 10.1186/s12889-017-4566-3

-

Article: https://bmcpublichealth.biomedcentral.com/articles/10.1186/s12889-017-4577-0

Publication date: 17 July 2017.

Correct citation: Tadesse F, Fogarty A, Deressa W. Prevalence and associated risk factors of malaria among adults in East Shewa Zone of Oromia Regional State, Ethiopia: a cross-sectional study. BMC Public Health. 2017;18:1. doi: 10.1186/s12889-017-4577-0

-

Article: https://bmcpublichealth.biomedcentral.com/articles/10.1186/s12889-017-4585-0

Publication date: 17 July 2017.

Correct citation: Hoek R, Havermans B, Houtman I, Brouwers E, Heerkens Y, Zijlstra-Vlasveld M et al. Stress Prevention@Work: a study protocol for the evaluation of a multifaceted integral stress prevention strategy to prevent employee stress in a healthcare organization: a cluster controlled trial. BMC Public Health. 2017;18:1. doi: 10.1186/s12889-017-4585-0

-

Article: https://bmcpublichealth.biomedcentral.com/articles/10.1186/s12889-017-4557-4

Publication date: 17 July 2017.

Correct citation: Douine M, Mosnier E, Le Hingrat Q, Charpentier C, Corlin F, Hureau L et al. Illegal gold miners in French Guiana: a neglected population with poor health. BMC Public Health. 2017;18:1. doi: 10.1186/s12889-017-4557-4

-

Article: https://bmcpublichealth.biomedcentral.com/articles/10.1186/s12889-017-4584-1

Publication date: 18 July 2017.

Correct citation: Rich P, Aarons G, Takemoto M, Cardenas V, Crist K, Bolling K et al. Implementation-effectiveness trial of an ecological intervention for physical activity in ethnically diverse low income senior centers. BMC Public Health. 2017;18:1. doi: 10.1186/s12889-017-4584-1

-

Article: https://bmcpublichealth.biomedcentral.com/articles/10.1186/s12889-017-4589-9

Publication date: 18 July 2017.

Correct citation: Kwesiga B, Pande G, Ario A, Tumwesigye N, Matovu J, Zhu B. A prolonged, community-wide cholera outbreak associated with drinking water contaminated by sewage in Kasese District, western Uganda. BMC Public Health. 2017;18:1. doi: 10.1186/s12889-017-4589-9

-

Article: https://bmcpublichealth.biomedcentral.com/articles/10.1186/s12889-017-4591-2

Publication date: 18 July 2017.

Correct citation: Seif S, kohi T, Moshiro C. Caretaker-adolescent communication on sexual and reproductive health: a cross-sectional study in Unguja-Tanzania Zanzibar. BMC Public Health. 2017;18:1. doi: 10.1186/s12889-017-4591-2

-

Article: https://bmcpublichealth.biomedcentral.com/articles/10.1186/s12889-017-4581-4

Publication date: 18 July 2017.

Correct citation: Victor M, Lau B, Ruud T. Predictors of return to work among patients in treatment for common mental disorders: a pre-post study. BMC Public Health. 2017;18:1. doi: 10.1186/s12889-017-4581-4

-

Article: https://bmcpublichealth.biomedcentral.com/articles/10.1186/s12889-017-4586-z

Publication date: 18 July 2017.

Correct citation: Kushnir V, Selby P, Zawertailo L, Tyndale R, Leatherdale S, Cunningham J. Long-term effectiveness of mailed nicotine replacement therapy: study protocol of a randomized controlled trial 5-year follow-up. BMC Public Health. 2017;18:1. doi: 10.1186/s12889-017-4586-z

-

Article: https://bmcpublichealth.biomedcentral.com/articles/10.1186/s12889-017-4582-3

Publication date: 19 July 2017.

Correct citation: de Oliveira Santos R, Vieira D, Miranda A, Fisberg R, Marchioni D, Baltar V. The traditional lunch pattern is inversely correlated with body mass index in a population-based study in Brazil. BMC Public Health. 2017;18:1. doi: 10.1186/s12889-017-4582-3

-

Article: https://bmcpublichealth.biomedcentral.com/articles/10.1186/s12889-017-4597-9

Publication date: 19 July 2017.

Correct citation: Shayo G, Chitama D, Moshiro C, Aboud S, Bakari M, Mugusi F. Cost-Effectiveness of isoniazid preventive therapy among HIV-infected patients clinicaly screened for latent tuberculosis infection in Dar es Salaam, Tanzania: A prospective Cohort study. BMC Public Health. 2017;18:1. doi: 10.1186/s12889-017-4597-9

-

Article: https://bmcpublichealth.biomedcentral.com/articles/10.1186/s12889-017-4598-8

Publication date: 19 July 2017.

Correct citation: Patel O, Shahulhameed S, Shivashankar R, Tayyab M, Rahman A, Prabhakaran D et al. Association between full service and fast food restaurant density, dietary intake and overweight/obesity among adults in Delhi, India. BMC Public Health. 2017;18:1. doi: 10.1186/s12889-017-4598-8

-

Article: https://bmcpublichealth.biomedcentral.com/articles/10.1186/s12889-017-4576-1

Publication date: 19 July 2017.

Correct citation: Amaral M, Herrin W, Gulere G. Using the Uganda National Panel Survey to analyze the effect of staple food consumption on undernourishment in Ugandan children. BMC Public Health. 2017;18:1. doi: 10.1186/s12889-017-4576-1

-

Article: https://bmcpublichealth.biomedcentral.com/articles/10.1186/s12889-017-4590-3

Publication date: 19 July 2017.

Correct citation: Kõks G, Fischer K, Kõks S. Smoking-related general and cause-specific mortality in Estonia. BMC Public Health. 2017;18:1. doi: 10.1186/s12889-017-4590-3

-

Article https://bmcpublichealth.biomedcentral.com/articles/10.1186/s12889-017-4583-2

Publication date: 20 July 2017.

Correct citation: Manana P, Kuonza L, Musekiwa A, Mpangane H, Koekemoer L. Knowledge, attitudes and practices on malaria transmission in Mamfene, KwaZulu-Natal Province, South Africa 2015. BMC Public Health. 2017;18:1. doi: 10.1186/s12889-017-4583-2

-

Article https://bmcpublichealth.biomedcentral.com/articles/10.1186/s12889-017-4570-7

Publication date: 20 July 2017.

Correct citation: de Wit L, Fenenga C, Giammarchi C, di Furia L, Hutter I, de Winter A et al. Community-based initiatives improving critical health literacy: a systematic review and meta-synthesis of qualitative evidence. BMC Public Health. 2017;18:1. doi: 10.1186/s12889-017-4570-7

-

Article https://bmcpublichealth.biomedcentral.com/articles/10.1186/s12889-017-4594-z

Publication date: 20 July 2017.

Correct citation: Begen F, Barnett J, Barber M, Payne R, Gowland M, Lucas J. Parents’ and caregivers’ experiences and behaviours when eating out with children with a food hypersensitivity. BMC Public Health. 2017;18:1. doi: 10.1186/s12889-017-4594-z

-

Article https://bmcpublichealth.biomedcentral.com/articles/10.1186/s12889-017-4601-4

Publication date: 20 July 2017.

Correct citation: Fan X, Zhou Z, Dang S, Xu Y, Gao J, Zhou Z et al. Exploring status and determinants of prenatal and postnatal visits in western China: in the background of the new health system reform. BMC Public Health. 2017;18:1. doi: 10.1186/s12889-017-4601-4

-

Article https://bmcpublichealth.biomedcentral.com/articles/10.1186/s12889-017-4587-y

Publication date: 21 July 2017.

Correct citation: Schwarzinger M, Thiébaut S, Baillot S, Mallet V, Rehm J. Alcohol use disorders and associated chronic disease – a national retrospective cohort study from France. BMC Public Health. 2017;18:1. doi: 10.1186/s12889-017-4587-y

-

Article https://bmcpublichealth.biomedcentral.com/articles/10.1186/s12889-017-4595-y

Publication date: 21 July 2017.

Correct citation: Mihrshahi S, Drayton B, Bauman A, Hardy L. Associations between childhood overweight, obesity, abdominal obesity and obesogenic behaviors and practices in Australian homes. BMC Public Health. 2017;18:1. doi: 10.1186/s12889-017-4595-y

-

Article https://bmcpublichealth.biomedcentral.com/articles/10.1186/s12889-017-4593-0

Publication date: 21 July 2017.

Correct citation: Maughan-Brown B, Venkataramani A. Accuracy and determinants of perceived HIV risk among young women in South Africa. BMC Public Health. 2017;18:1. doi: 10.1186/s12889-017-4593-0

-

Article https://bmcpublichealth.biomedcentral.com/articles/10.1186/s12889-017-4569-0

Publication date: 24 July 2017.

Correct citation: Eliott J, Forster A, McDonough J, Bowd K, Crabb S. An examination of Australian newspaper coverage of the link between alcohol and cancer 2005 to 2013. BMC Public Health. 2017;18:1. doi: 10.1186/s12889-017-4569-0

-

Article https://bmcpublichealth.biomedcentral.com/articles/10.1186/s12889-017-4509-z

Publication date: 24 July 2017.

Correct citation: Hambidge K, Krebs N, Garcés A, Westcott J, Figueroa L, Goudar S et al. Anthropometric indices for non-pregnant women of childbearing age differ widely among four low-middle income populations. BMC Public Health. 2017;18:1. doi: 10.1186/s12889-017-4509-z

-

Article https://bmcpublichealth.biomedcentral.com/articles/10.1186/s12889-017-4599-7

Publication date: 24 July 2017.

Correct citation: Helou K, El Helou N, Mahfouz M, Mahfouz Y, Salameh P, Harmouche-Karaki M. Validity and reliability of an adapted arabic version of the long international physical activity questionnaire. BMC Public Health. 2017;18:1. doi: 10.1186/s12889-017-4599-7

-

Article https://bmcpublichealth.biomedcentral.com/articles/10.1186/s12889-017-4603-2

Publication date: 24 July 2017.

Correct citation: Shepherd S, Delgado R, Sherwood J, Paradies Y. The impact of indigenous cultural identity and cultural engagement on violent offending. BMC Public Health. 2017;18:1. doi: 10.1186/s12889-017-4603-2

-

Article https://bmcpublichealth.biomedcentral.com/articles/10.1186/s12889-017-4579-y

Publication date: 24 July 2017.

Correct citation: Sreeramareddy C, Ramakrishnareddy N. Association of adult tobacco use with household food access insecurity: results from Nepal demographic and health survey, 2011. BMC Public Health. 2017;18:1. doi: 10.1186/s12889-017-4579-y

-

Article https://bmcpublichealth.biomedcentral.com/articles/10.1186/s12889-017-4532-0

Publication date: 24 July 2017.

Correct citation: Johnsen N, Davidsen M, Michelsen S, Juel K. Health profile for Danish adults with activity limitation: a cross-sectional study. BMC Public Health. 2017;18:1. doi: 10.1186/s12889-017-4532-0

-

Article https://bmcpublichealth.biomedcentral.com/articles/10.1186/s12889-017-4596-x

Publication date: 25 July 2017.

Correct citation: Pärna K, Põld M, Ringmets I. Trends in smoking behaviour among Estonian physicians in 1982–2014. BMC Public Health. 2017;18:1. doi: 10.1186/s12889-017-4596-x

-

Article https://bmcpublichealth.biomedcentral.com/articles/10.1186/s12889-017-4563-6

Publication date: 25 July 2017.

Correct citation: Liang Y, Wang Y, Li Z, He L, Xu Y, Zhang Q et al. Caregiving burden and depression in paid caregivers of hospitalized patients: a pilot study in China. BMC Public Health. 2017;18:1. doi: 10.1186/s12889-017-4563-6

-

Article https://bmcpublichealth.biomedcentral.com/articles/10.1186/s12889-017-4558-3

Publication date: 25 July 2017.

Correct citation: Wan X, Ren H, Ma E, Yang G. Mortality trends for ischemic heart disease in China: an analysis of 102 continuous disease surveillance points from 1991 to 2009. BMC Public Health. 2017;18:1. doi: 10.1186/s12889-017-4558-3

-

Article https://bmcpublichealth.biomedcentral.com/articles/10.1186/s12889-017-4611-2

Publication date: 25 July 2017.

Correct citation: Chadambuka A, Katirayi L, Muchedzi A, Tumbare E, Musarandega R, Mahomva A et al. Acceptability of lifelong treatment among HIV-positive pregnant and breastfeeding women (Option B+) in selected health facilities in Zimbabwe: a qualitative study. BMC Public Health. 2017;18:1. doi: 10.1186/s12889-017-4611-2

-

Article https://bmcpublichealth.biomedcentral.com/articles/10.1186/s12889-017-4617-9

Publication date: 25 July 2017.

Correct citation: Ali N, Mahmood S, Manirujjaman M, Perveen R, Al Nahid A, Ahmed S et al. Hypertension prevalence and influence of basal metabolic rate on blood pressure among adult students in Bangladesh. BMC Public Health. 2017;18:1. doi: 10.1186/s12889-017-4617-9

-

Article https://bmcpublichealth.biomedcentral.com/articles/10.1186/s12889-017-4592-1

Publication date: 25 July 2017.

Correct citation: Teuscher D, Bukman A, van Baak M, Feskens E, Renes R, Meershoek A. A lifestyle intervention study targeting individuals with low socioeconomic status of different ethnic origins: important aspects for successful implementation. BMC Public Health. 2017;18:1.. doi: 10.1186/s12889-017-4592-1

-

Article https://bmcpublichealth.biomedcentral.com/articles/10.1186/s12889-017-4610-3

Publication date: 25 July 2017.

Correct citation: Shonkoff E, Anzman-Frasca S, Lynskey V, Chan G, Glenn M, Economos C. Child and parent perspectives on healthier side dishes and beverages in restaurant kids’ meals: results from a national survey in the United States. BMC Public Health. 2017;18:1. doi: 10.1186/s12889-017-4610-3

-

Article https://bmcpublichealth.biomedcentral.com/articles/10.1186/s12889-017-4620-1

Publication date: 25 July 2017.

Correct citation: Grunseit A, Richards J, Merom D. Running on a high: parkrun and personal well-being. BMC Public Health. 2017;18:1. doi: 10.1186/s12889-017-4620-1

-

Article https://bmcpublichealth.biomedcentral.com/articles/10.1186/s12889-017-4549-4

Publication date: 26 July 2017.

Correct citation: Lazarus J, Sperle I, Safreed-Harmon K, Gore C, Cebolla B, Spina A. Associations between national viral hepatitis policies/programmes and country-level socioeconomic factors: a sub-analysis of data from the 2013 WHO viral hepatitis policy report. BMC Public Health. 2017;18:1. doi: 10.1186/s12889-017-4549-4

-

Article https://bmcpublichealth.biomedcentral.com/articles/10.1186/s12889-017-4609-9

Publication date: 26 July 2017.

Correct citation: Hakeberg M, Wide Boman U. Self-reported oral and general health in relation to socioeconomic position. BMC Public Health. 2017;18:1. doi: 10.1186/s12889-017-4609-9

-

Article https://bmcpublichealth.biomedcentral.com/articles/10.1186/s12889-017-4602-3

Publication date: 26 July 2017.

Correct citation: Peetoom K, Crutzen R, Bohnen J, Verhoeven R, Nelissen-Vrancken H, Winkens B et al. Optimising decision making on illness absenteeism due to fever and common infections within childcare centres: development of a multicomponent intervention and study protocol of a cluster randomised controlled trial. BMC Public Health. 2017;18:1. doi: 10.1186/s12889-017-4602-3

-

Article https://bmcpublichealth.biomedcentral.com/articles/10.1186/s12889-017-4605-0

Publication date: 26 July 2017.

Correct citation: Harding-Esch E, Kadimpeul J, Sarr B, Sane A, Badji S, Laye M et al. Population-based prevalence survey of follicular trachoma and trachomatous trichiasis in the Casamance region of Senegal. BMC Public Health. 2017;18:1. doi: 10.1186/s12889-017-4605-0

-

Article https://bmcpublichealth.biomedcentral.com/articles/10.1186/s12889-017-4443-0

Publication date: 26 July 2017.

Correct citation: Batista M, Lawrence H, Sousa M. Oral health literacy and oral health outcomes in an adult population in Brazil. BMC Public Health. 2017;18:1. doi: 10.1186/s12889-017-4443-0

-

Article https://bmcpublichealth.biomedcentral.com/articles/10.1186/s12889-017-4622-z

Publication date: 26 July 2017.

Correct citation: Gamage A, Jayawardana P. Knowledge of non-communicable diseases and practices related to healthy lifestyles among adolescents, in state schools of a selected educational division in Sri Lanka. BMC Public Health. 2017;18:1. doi: 10.1186/s12889-017-4622-z

-

Article https://bmcpublichealth.biomedcentral.com/articles/10.1186/s12889-017-4614-z

Publication date: 28 July 2017.

Correct citation: Yang X, Feldman M. A reversed gender pattern? A meta-analysis of gender differences in the prevalence of non-suicidal self-injurious behaviour among Chinese adolescents. BMC Public Health. 2017;18:1. doi: 10.1186/s12889-017-4614-z

-

Article https://bmcpublichealth.biomedcentral.com/articles/10.1186/s12889-017-4608-x

Publication date: 28 July 2017.

Correct citation: Chung P, Zhang C, Liu J, Chan D, Si G, Hagger M. The process by which perceived autonomy support predicts motivation, intention, and behavior for seasonal influenza prevention in Hong Kong older adults. BMC Public Health. 2017;18:1. doi: 10.1186/s12889-017-4608-x

-

Article https://bmcpublichealth.biomedcentral.com/articles/10.1186/s12889-017-4613-0

Publication date: 1 August 2017.

Correct citation: Ward B, Kippen R, Munro G, Buykx P, McBride N, Wiggers J et al. Liquor licences issued to Australian schools. BMC Public Health. 2017;18:1. doi: 10.1186/s12889-017-4613-0

-

Article https://bmcpublichealth.biomedcentral.com/articles/10.1186/s12889-017-4638-4

Publication date: 1 August 2017.

Correct citation: Slekiene J, Mosler H. Does depression moderate handwashing in children?. BMC Public Health. 2017;18:1. doi: 10.1186/s12889-017-4638-4

-

Article https://bmcpublichealth.biomedcentral.com/articles/10.1186/s12889-017-4626-8

Publication date: 1 August 2017.

Correct citation: Howse E, Freeman B, Wu J, Rooney K. ‘The university should promote health, but not enforce it’: opinions and attitudes about the regulation of sugar-sweetened beverages in a university setting. BMC Public Health. 2017;18:1. doi: 10.1186/s12889-017-4626-8

-

Article https://bmcpublichealth.biomedcentral.com/articles/10.1186/s12889-017-4633-9

Publication date: 1 August 2017.

Correct citation: Churruca K, Mitchell R. Exploring coronial determination of intent for poisoning-related deaths in Australia, 2001–2013. BMC Public Health. 2017;18:1. doi: 10.1186/s12889-017-4633-9

-

Article https://bmcpublichealth.biomedcentral.com/articles/10.1186/s12889-017-4604-1

Publication date: 1 August 2017.

Correct citation: Herrmann A, Fischer H, Amelung D, Litvine D, Aall C, Andersson C et al. Household preferences for reducing greenhouse gas emissions in four European high-income countries: Does health information matter? A mixed-methods study protocol. BMC Public Health. 2017;18:1. doi: 10.1186/s12889-017-4604-1

-

Article https://bmcpublichealth.biomedcentral.com/articles/10.1186/s12889-017-4647-3

Publication date: 1 August 2017.

Correct citation: Hewett P, Austrian K, Soler-Hampejsek E, Behrman J, Bozzani F, Jackson-Hachonda N. Erratum to: cluster randomized evaluation of adolescent girls empowerment Programme (AGEP): study protocol. BMC Public Health. 2017;18:1. doi: 10.1186/s12889-017-4647-3

-

Article https://bmcpublichealth.biomedcentral.com/articles/10.1186/s12889-017-4641-9

Publication date: 1 August 2017.

Correct citation: Wuni C, Turpin C, Dassah E. Determinants of contraceptive use and future contraceptive intentions of women attending child welfare clinics in urban Ghana. BMC Public Health. 2017;18:1. doi: 10.1186/s12889-017-4641-9

-

Article https://bmcpublichealth.biomedcentral.com/articles/10.1186/s12889-017-4628-6

Publication date: 1 August 2017.

Correct citation: Lukaszyk C, Coombes J, Turner N, Hillmann E, Keay L, Tiedemann A et al. Yarning about fall prevention: community consultation to discuss falls and appropriate approaches to fall prevention with older Aboriginal and Torres Strait Islander people. BMC Public Health. 2017;18:1. doi: 10.1186/s12889-017-4628-6

-

Article https://bmcpublichealth.biomedcentral.com/articles/10.1186/s12889-017-4642-8

Publication date: 1 August 2017.

Correct citation: Bader R, Shihab R, Al-Rimawi D, Hawari F. Informing tobacco control policy in Jordan: assessing the effectiveness of pictorial warning labels on cigarette packs. BMC Public Health. 2017;18:1. doi: 10.1186/s12889-017-4642-8

-

Article https://bmcpublichealth.biomedcentral.com/articles/10.1186/s12889-017-4572-5

Publication date: 1 August 2017.

Correct citation: Indig D, Lee K, Grunseit A, Milat A, Bauman A. Pathways for scaling up public health interventions. BMC Public Health. 2017;18:1. doi: 10.1186/s12889-017-4572-5

-

Article https://bmcpublichealth.biomedcentral.com/articles/10.1186/s12889-017-4643-7

Publication date: 1 August 2017.

Correct citation: Alghafri T, Alharthi S, Al-farsi Y, Bannerman E, Craigie A, Anderson A. Correlates of physical activity and sitting time in adults with type 2 diabetes attending primary health care in Oman. BMC Public Health. 2017;18:1. doi: 10.1186/s12889-017-4643-7

-

Article https://bmcpublichealth.biomedcentral.com/articles/10.1186/s12889-017-4639-3

Publication date: 1 August 2017.

Correct citation: Idigoras I, Arrospide A, Portillo I, Arana-Arri E, Martínez-Indart L, Mar J et al. Evaluation of the colorectal cancer screening Programme in the Basque Country (Spain) and its effectiveness based on the Miscan-colon model. BMC Public Health. 2017;18:1. doi: 10.1186/s12889-017-4639-3

-

Article https://bmcpublichealth.biomedcentral.com/articles/10.1186/s12889-017-4621-0

Publication date: 1 August 2017.

Correct citation: Kismul H, Acharya P, Mapatano M, Hatløy A. Determinants of childhood stunting in the Democratic Republic of Congo: further analysis of Demographic and Health Survey 2013–14. BMC Public Health. 2017;18:1. doi: 10.1186/s12889-017-4621-0

-

Article https://bmcpublichealth.biomedcentral.com/articles/10.1186/s12889-017-4607-y

Publication date: 1 August 2017.

Correct citation: Andersen M, Williamson A, Fernando P, Wright D, Redman S. Housing conditions of urban households with Aboriginal children in NSW Australia: tenure type matters. BMC Public Health. 2017;18:1. doi: 10.1186/s12889-017-4607-y

-

Article https://bmcpublichealth.biomedcentral.com/articles/10.1186/s12889-017-4634-8

Publication date: 1 August 2017.

Correct citation: Osborne J, Wilson C, Duncan A, Cole S, Flight I, Turnbull D et al. Patterns of participation over four rounds of annual fecal immunochemical test-based screening for colorectal cancer: what predicts rescreening?. BMC Public Health. 2017;18:1. doi: 10.1186/s12889-017-4634-8

-

Article https://bmcpublichealth.biomedcentral.com/articles/10.1186/s12889-017-4573-4

Publication date: 1 August 2017.

Correct citation: Barnish M, Morgan H, Barnish J. The 2016 HIGh Heels: Health effects And psychosexual BenefITS (HIGH HABITS) study: systematic review of reviews and additional primary studies. BMC Public Health. 2017;18:1. doi: 10.1186/s12889-017-4573-4

-

Article https://bmcpublichealth.biomedcentral.com/articles/10.1186/s12889-017-4612-1

Publication date: 1 August 2017.

Correct citation: Saunders N, Macpherson A, Guan J, Sheng L, Guttmann A. The shrinking health advantage: unintentional injuries among children and youth from immigrant families. BMC Public Health. 2017;18:1. doi: 10.1186/s12889-017-4612-1

-

Article https://bmcpublichealth.biomedcentral.com/articles/10.1186/s12889-017-4600-5

Publication date: 1 August 2017.

Correct citation: Berglind D, Tynelius P. Objectively measured physical activity patterns, sedentary time and parent-reported screen-time across the day in four-year-old Swedish children. BMC Public Health. 2017;18:1. doi: 10.1186/s12889-017-4600-5

-

Article https://bmcpublichealth.biomedcentral.com/articles/10.1186/s12889-017-4624-x

Publication date: 1 August 2017.

Correct citation: Decker M, Tomko C, Wingo E, Sawyer A, Peitzmeier S, Glass N et al. A brief, trauma-informed intervention increases safety behavior and reduces HIV risk for drug-involved women who trade sex. BMC Public Health. 2017;18:1. doi: 10.1186/s12889-017-4624-x

-

Article https://bmcpublichealth.biomedcentral.com/articles/10.1186/s12889-017-4631-y

Publication date: 1 August 2017.

Correct citation: Awua A, Wiredu E, Afari E, Tijani A, Djanmah G, Adanu R. A tailored within-community specimen collection strategy increased uptake of cervical cancer screening in a cross-sectional study in Ghana. BMC Public Health. 2017;18:1. doi: 10.1186/s12889-017-4631-y

-

Article https://bmcpublichealth.biomedcentral.com/articles/10.1186/s12889-017-4632-x

Publication date: 2 August 2017.

Correct citation: Yu Y, Hu S, Yang Y, Zhao X, Xue J, Zhang J et al. Successive monitoring surveys of selected banned and restricted pesticide residues in vegetables from the northwest region of China from 2011 to 2013. BMC Public Health. 2017;18:1. doi: 10.1186/s12889-017-4632-x

-

Article https://bmcpublichealth.biomedcentral.com/articles/10.1186/s12889-017-4616-x

Publication date: 2 August 2017.

Correct citation: Liu T, Zhu G, He J, Song T, Zhang M, Lin H et al. Early rigorous control interventions can largely reduce dengue outbreak magnitude: experience from Chaozhou, China. BMC Public Health. 2017;18:1.. doi: 10.1186/s12889-017-4616-x

-

Article https://bmcpublichealth.biomedcentral.com/articles/10.1186/s12889-017-4615-y

Publication date: 2 August 2017.

Correct citation: Hsu J, Chang S, Lu C. Geographic Variations and Time Trends in Cancer Treatments in Taiwan. BMC Public Health. 2017;18:1. doi: 10.1186/s12889-017-4615-y

-

Article https://bmcpublichealth.biomedcentral.com/articles/10.1186/s12889-017-4574-3

Publication date: 2 August 2017.

Correct citation: Seppälä T, Hankonen N, Korkiakangas E, Ruusuvuori J, Laitinen J. National policies for the promotion of physical activity and healthy nutrition in the workplace context: a behaviour change wheel guided content analysis of policy papers in Finland. BMC Public Health. 2017;18:1. doi: 10.1186/s12889-017-4574-3

-

Article https://bmcpublichealth.biomedcentral.com/articles/10.1186/s12889-017-4562-7

Publication date: 2 August 2017.

Correct citation: Ghosn W, Menvielle G, Rican S, Rey G. Associations of cause-specific mortality with area level deprivation and travel time to health care in France from 1990 to 2007, a multilevel analysis. BMC Public Health. 2017;18:1. doi: 10.1186/s12889-017-4562-7

-

Article https://bmcpublichealth.biomedcentral.com/articles/10.1186/s12889-017-4606-z

Publication date: 2 August 2017.

Correct citation: Almahdi H, Ali R, Nasir E, Åstrøm A. Socio-cognitive correlates of intention to use Toombak: a cross-sectional study among students (13–16 years) in Khartoum State, Sudan. BMC Public Health. 2017;18:1. doi: 10.1186/s12889-017-4606-z

-

Article https://bmcpublichealth.biomedcentral.com/articles/10.1186/s12889-017-4644-6

Publication date: 3 August 2017.

Correct citation: Shysh A, Nguyen L, Guo M, Vaska M, Naugler C, Rashid-Kolvear F. The incidence of acute myeloid leukemia in Calgary, Alberta, Canada: a retrospective cohort study. BMC Public Health. 2017;18:1. doi: 10.1186/s12889-017-4644-6

-

Article https://bmcpublichealth.biomedcentral.com/articles/10.1186/s12889-017-4651-7

Publication date: 3 August 2017.

Correct citation: Wu C, Zhu X, Kang Y, Cao Y, Lu P, Zhou W et al. Epidemiology of Humanpapilloma virus infection among women in Fujian, China. BMC Public Health. 2017;18:1. doi: 10.1186/s12889-017-4651-7

-

Article https://bmcpublichealth.biomedcentral.com/articles/10.1186/s12889-017-4568-1

Publication date: 3 August 2017.

Correct citation: Mactaggart F, McDermott L, Tynan A, Gericke C. Exploring the determinants of health and wellbeing in communities living in proximity to coal seam gas developments in regional Queensland. BMC Public Health. 2017;18:1. doi: 10.1186/s12889-017-4568-1

-

Article https://bmcpublichealth.biomedcentral.com/articles/10.1186/s12889-017-4640-x

Publication date: 3 August 2017.

Correct citation: Ehrlich R, Montgomery A, Akugizibwe P, Gonsalves G. Public health implications of changing patterns of recruitment into the South African mining industry, 1973–2012: a database analysis. BMC Public Health. 2017;18:1. doi: 10.1186/s12889-017-4640-x

-

Article https://bmcpublichealth.biomedcentral.com/articles/10.1186/s12889-017-4652-6

Publication date: 3 August 2017.

Correct citation: Yeboah K, Dodam K, Affrim P, Adu-Gyamfi L, Bado A, Owusu Mensah R et al. Metabolic syndrome and parental history of cardiovascular disease in young adults in urban Ghana. BMC Public Health. 2017;18:1. doi: 10.1186/s12889-017-4652-6

-

Article https://bmcpublichealth.biomedcentral.com/articles/10.1186/s12889-017-4625-9

Publication date: 3 August 2017.

Correct citation: Waters E, Gibbs L, Tadic M, Ukoumunne O, Magarey A, Okely A et al. Cluster randomised trial of a school-community child health promotion and obesity prevention intervention: findings from the evaluation of fun ‘n healthy in Moreland!. BMC Public Health. 2017;18:1. doi: 10.1186/s12889-017-4625-9

Since this error was detected the journal has resumed publication in the 2017 volume, volume number 17. In 2018 the journal will resume publication in volume number 18. The publisher apologizes for any confusion caused by this error.

